# Neuromuscular training with injury prevention counselling to decrease the risk of acute musculoskeletal injury in young men during military service: a population-based, randomised study

**DOI:** 10.1186/1741-7015-9-35

**Published:** 2011-04-11

**Authors:** Jari Parkkari, Henri Taanila, Jaana Suni, Ville M Mattila, Olli Ohrankämmen, Petteri Vuorinen, Pekka Kannus, Harri Pihlajamäki

**Affiliations:** 1 Tampere Research Centre of Sports Medicine, UKK Institute, P.O. Box 30, 33501 Tampere, Finland; 2 Research Department, Centre for Military Medicine, P.O. Box 2, 15701 Lahti, Finland; 3 General Headquarters of Finnish Defence Forces, P.O. Box 919, 00131 Helsinki, Finland; 4 Staff Department, Pori Brigade, P.O. Box 38, 27801 Säkylä, Finland; 5 Research Unit of Pirkanmaa Hospital District and Division of Orthopaedics and Traumatology, Department of Trauma, Musculoskeletal Surgery and Rehabilitation, Tampere University Hospital, P.O Box 2000, 33521 Tampere, Finland

## Abstract

**Background:**

The rapidly increasing number of activity-induced musculoskeletal injuries among adolescents and young adults is currently a true public health burden. The objective of this study was to investigate whether a neuromuscular training programme with injury prevention counselling is effective in preventing acute musculoskeletal injuries in young men during military service.

**Methods:**

The trial design was a population-based, randomised study. Two successive cohorts of male conscripts in four companies of one brigade in the Finnish Defence Forces were first followed prospectively for one 6-month term to determine the baseline incidence of injury. After this period, two new successive cohorts in the same four companies were randomised into two groups and followed prospectively for 6 months. Military service is compulsory for about 90% of 19-year-old Finnish men annually, who comprised the cohort in this study. This randomised, controlled trial included 968 conscripts comprising 501 conscripts in the intervention group and 467 conscripts in the control group. A neuromuscular training programme was used to enhance conscripts' motor skills and body control, and an educational injury prevention programme was used to increase knowledge and awareness of acute musculoskeletal injuries. The main outcome measures were acute injuries of the lower and upper limbs.

**Results:**

In the intervention groups, the risk for acute ankle injury decreased significantly compared to control groups (adjusted hazards ratio (HR) = 0.34, 95% confidence interval (95% CI) = 0.15 to 0.78, *P *= 0.011). This risk decline was observed in conscripts with low as well as moderate to high baseline fitness levels. In the latter group of conscripts, the risk of upper-extremity injuries also decreased significantly (adjusted HR = 0.37, 95% CI 0.14 to 0.99, *P *= 0.047). In addition, the intervention groups tended to have less time loss due to injuries (adjusted HR = 0.55, 95% CI 0.29 to 1.04).

**Conclusions:**

A neuromuscular training and injury prevention counselling programme was effective in preventing acute ankle and upper-extremity injuries in young male army conscripts. A similar programme could be useful for all young individuals by initiating a regular exercise routine.

**Trial registration:**

ClinicalTrials.gov identifier number NCT00595816.

## Background

Current public health recommendations strongly suggest regular physical activity to improve cardiovascular health and reduce the risk of chronic diseases [1,2]. The risk of musculoskeletal injury also increases, however, with an increase in physical activity. The rapidly increasing number of activity-induced injuries among adolescents and young adults is currently considered a true public health burden [3,4].

Because of their anatomic location, the ankle and knee joints are subjected to tremendous force during exercise and physical activity. Thus, it is not surprising that they are the most common sites for injuries, usually accounting for 50% to 60% of all sports injuries [5,6]. Acute injuries of the limbs, especially those affecting the ankle, knee and shoulder joints, may also have long-term consequences. Ankle injuries recur easily [7-9], and severe knee injuries often lead to early osteoarthritis [10,11].

Several studies have demonstrated that a neuromuscular training programme can reduce the risk of ankle and knee injuries in athletes [12-22]. To our knowledge, the possibility of preventing injuries in a general population, such as in young individuals with various physical fitness levels, has not been assessed. Therefore, the aim of the present study was to investigate whether a systematic neuromuscular training and injury prevention counselling programme could reduce the risk of acute injury in young Finnish men.

## Methods

### Sample size

On the basis of previous studies of physical activity-related injuries [4,23], the incidence of acute lower-limb injuries was estimated to be 0.6 injuries per person-year. The power calculations were based on a negative binomial model with an assumption of overdispersion parameter of 1.50. Thus, a minimum 33% reduction in the incidence of lower-limb injuries, from 0.6 injuries per person-year in the control group to 0.4 injuries per person-year in the intervention group, would be detected with the sample size of 500 persons per group. The statistical power level was set to 0.80, and the statistical significance level was set at 0.05.

### Participants and randomisation

The participants of this study comprised male conscripts from four companies of one brigade (Pori Brigade, Säkylä, Finland) in the Finnish Defence Forces. The Pori Brigade is a typical Finnish garrison, and the chosen companies formed a representative sample of conscripts. Annually, the conscripts of each age cohort are randomly assigned into the companies.

The four companies enrolled into the study were the anti-tank company, the signal company, the mortar company and the engineer company. Military service in Finland is compulsory, and annually about 90% of 19-year-old men enter into the service. The service period varies from 6 to 12 months.

During the study, four cohorts of conscripts started service in the brigade: 359 in July 2006, 619 in January 2007, 522 in July 2007 and 557 in January 2008 (a total of 2,057 conscripts). The first two successive cohorts were followed prospectively for one term (6 months) to assess the baseline incidence of injuries (prestudy period) and to find out possible differences in the risk of acute injury in the participating companies. After this step, the four companies were randomised into two groups (two intervention companies and two control companies), and their two new successive cohorts were followed prospectively for one term, providing the data for the intervention.

**Figure 1:**
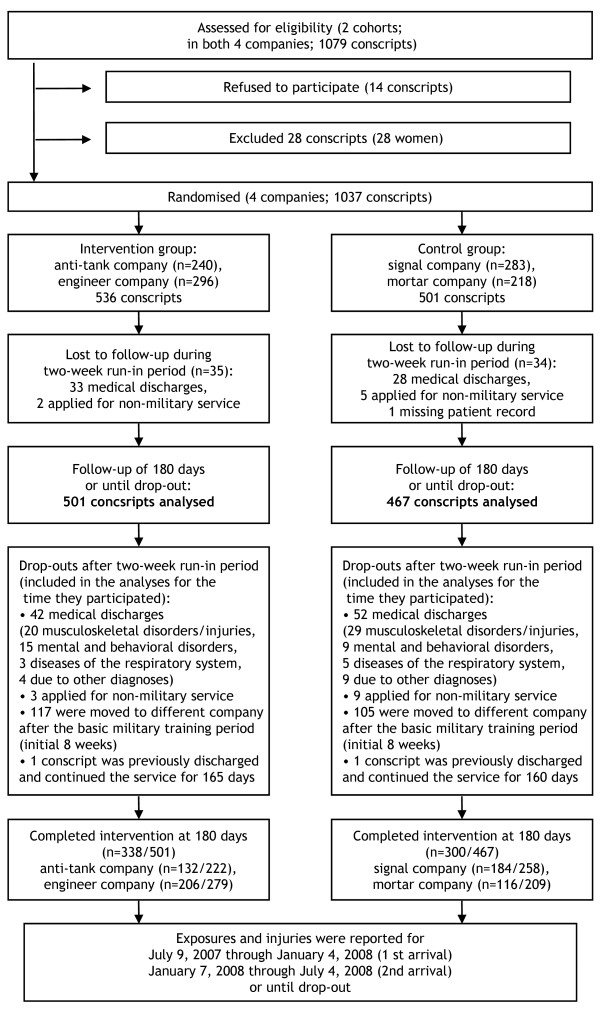
**Flowchart of companies and participants through the study during the randomised intervention**.

Eighteen conscripts during the prestudy period and fourteen during the study period refused to participate in the study. Therefore, 2,025 conscripts (98%) agreed to participate and provided their informed consent prior to the initiation of the study. Details of the flow of participants during the randomised intervention are shown in Figure 1.

The health status of conscripts was checked during the first 2 weeks of the study (run-in period) by routine medical screenings performed by a physician. During the intervention, 61 participants were lost to follow-up for medical reasons: 14 were permanently discharged from military service, and 47 were temporarily discharged for at least 6 months. Because there were only 28 women in the study (3%), their data were excluded from the analysis. Seven conscripts applied for nonmilitary service during the 2-week run-in period, and they were also excluded. Additionally, two conscripts were lost to follow-up because of a missing patient record, and one conscript applied for postponement of service during the run-in period.

Thus, during the intervention (study period), there were 501 and 467 conscripts in the intervention and control groups, respectively, eligible for analyses. Corresponding figures for the prestudy period were 508 and 436. The ages of the conscripts ranged from 18 to 28 years (median and mean age 19 years). The baseline characteristics of the study subjects in the four companies were stratified into two study periods, and these are presented in Table 1. There were some statistically significant differences between the companies, and thus these variables were adjusted in the statistical models.

**Table 1:** Baseline characteristics of 1,912 male conscripts by company and study period

Variable	Prestudy period				Study period intervention groups		Study period control group		Missing data	*P *value^a^
			
	Anti-tank company	Engineer company	Signal company	Mortar company	Anti-tank company	Engineer company	Signal company	Mortar company		
Number of conscripts	263	245	282	154	222	279	258	209	0 (0%)	-
Median age, yr	19	19	19	19	19	19	19	19	0 (0%)	0.054^b^
Median body mass index, kg/m^2^	23.4	23.6	22.5	22.7	23.6	23.3	22.8	23.7	175 (9%)	0.011^b^
Median waist circumference, cm	87.0	87.0	85.0	84.5	85.0	86.0	84.0	86.1	139 (7%)	0.005^b^
Median 12-minute running test result, m	2,310	2,400	2,340	2,515	2,350	2,420	2,300	2,470	51 (3%)	0.614^b^
Median muscle fitness index^d^, points	7	7	7	8	7	6	6	10	37 (2%)	0.019^b^
Median conscript physical fitness index (CPFI)^e^, points	15.05	15.50	15.03	16.75	15.75	15.25	14.60	17.05	58 (3%)	0.153^b^
Conscript's hometown population ≥10,000, %	59	57	64	54	66	57	68	63	25 (1%)	0.100^c^
High level of preceding physical activity^f^, %	31	36	26	32	24	26	21	49	24 (1%)	0.011^c^
Good self-assessed health^g^, %	57	51	54	50	54	53	41	70	23 (1%)	0.942^c^
Chronic impairment or disability, %	17	17	11	17	11	18	19	16	30 (2%)	0.277^c^
Past orthopaedic surgery, %	8	9	7	9	9	10	11	7	25 (1%)	0.802^c^
No musculoskeletal symptoms^h^, %	28	27	32	28	34	34	31	25	25 (1%)	0.143^c^
Previous or current regular smoker, %	43	57	47	40	53	58	47	46	27 (1%)	0.003^c^
Use of alcohol at least three times per week, %	16	20	15	16	24	23	23	14	24 (1%)	0.010^c^

^a^*P *value for difference between the study group and study year; ^b^*P *value was calculated by using a Kruskal-Wallis test for median difference; ^c^*P *value was calculated by using χ^2 ^statistics for significant differences; ^d^Muscle fitness index is the sum of individual muscle fitness test results comprising pushups, situps, pullups, the standing long jump and the back-lift test (excellent = 13 to 15 points, good = 9 to 12 points, fair to good = 5 to 8 points, and poor = 0 to 4 points); ^e^CPFI = (12-minute running test result (measured in meters) + 100 × muscle fitness index) ÷ 200; scoring was excellent = CPFI ≥21.00), good = 17.00 ≤ CPFI < 21.00, fair to good = 13.00 ≤ CPFI < 17.00, and poor = CPFI < 13.00; ^f^sweating exercise at least three times per week during the past month before entry into the military; ^g^compared to age cohort; ^h^symptoms lasting more than 7 days in at least one anatomical region during the past month before entering the military.

Using the company as the unit of randomisation with a computer-generated randomisation programme, an independent statistician who had no information about the study subjects performed the randomisation of companies into the intervention and control groups for the July 2007 and January 2008 cohorts. Companies allocated to the intervention group were informed about the upcoming programme for preventing injuries. Companies in the control group followed the usual regimen of the Finnish army.

All subjects were followed for 6 months starting from the first day of service. If a conscript changed his company during the study, he was followed until the change took place, and this change was taken into account when calculating exposure times. Approval for the study protocol was obtained from the Ethics Committee of Pirkanmaa Hospital District (reference R07076). The clinical trial identification number is NCT00595816.

### Preinformation questionnaire

Subjects were administered a preinformation questionnaire during the first week of military service. Questions charted conscripts' socioeconomic factors, health and health behaviour at the baseline of the study. The socioeconomic factors included education level, urbanisation level of the place of residence, school success (educational level and grades combined) and father's occupational group. Health factors included previous sports injuries and orthopaedic surgeries, medications, chronic disease, chronic impairment or disability, self-assessed health compared to age mates and musculoskeletal pain in seven anatomical regions during the past month. Health behaviour was assessed on the basis of answers to questions about the use of alcohol and tobacco, frequency of drunkenness, amount of physical exercise, prior sporting activities, belonging to a sports club, participation in competitive sports, highest level achieved in school sports, self-assessed physical fitness and opinion about the physical demands on a soldier.

### Assessment of baseline physical fitness

A Cooper's test (12-minute running test) and muscular fitness tests were performed by most conscripts (97%) during their first 2 weeks of military service. A minority of conscripts (3%) were unable to complete their physical fitness tests because of minor health problems, such as infection or overuse injury. Muscular fitness tests and the 12-minute running test were performed on different days. Muscular fitness tests included pushups, situps, pullups, the standing long jump and a back-lift test [24]. A conscript's physical fitness index (CPFI) was calculated using the following formula: (12-minute running test result (measured in metres) + 100 × muscle fitness index) ÷ 200 (Table 1, footnotes d and e). The formula is based on standard practice in the Finnish Defence Forces since 1982 [25]. In addition, height, weight and waist circumference were measured during the first weeks of service. Body mass index (BMI) was calculated by dividing weight (in kilograms) by height (in meters squared). Waist circumference as a mark of abdominal obesity and excessive visceral fat [26] was measured using a tape measure midway between the lowest rib and the iliac crest after normal exhalation. The cutoff points for overweight and obesity on the basis of BMI and waist circumference were set according to the guidelines of the World Health Organisation [27].

### Basic physical training programme

At the beginning of military service, all conscripts performed 8 weeks of basic training, which consisted of various physical activities, including marching, cycling, skiing, orienteering, swimming, drill training and combat training or other training. Each week there were an average of 17 hours of military actions, with a gradual increase in intensity. During most of this time, the activity level was low to moderate in intensity. In addition, conscripts performed other physical exercises, such as jogging, team sports and circuit training for an average of 7 hours per week.

The 2-month basic training period was followed by 4-months specific military training programme, depending on the company and service duration. During this 6-month period of service, the amount and intensity of physical training was maintained at approximately the same level in different companies.

### Intervention programme

The intervention included neuromuscular training and injury prevention counselling with cognitive-behavioural learning goals. This programme was included in addition to the above-noted basic training. The main aim of this programme was to decrease the number of musculoskeletal injuries during military service. Implementation of the intervention was planned together with the personnel of the brigade as well as with conscripts in leading positions. Two educated female instructors outside the brigade, one of whom had completed military service, were responsible for conducting the implementation of the intervention.

#### Neuromuscular training

**Figure 2:**
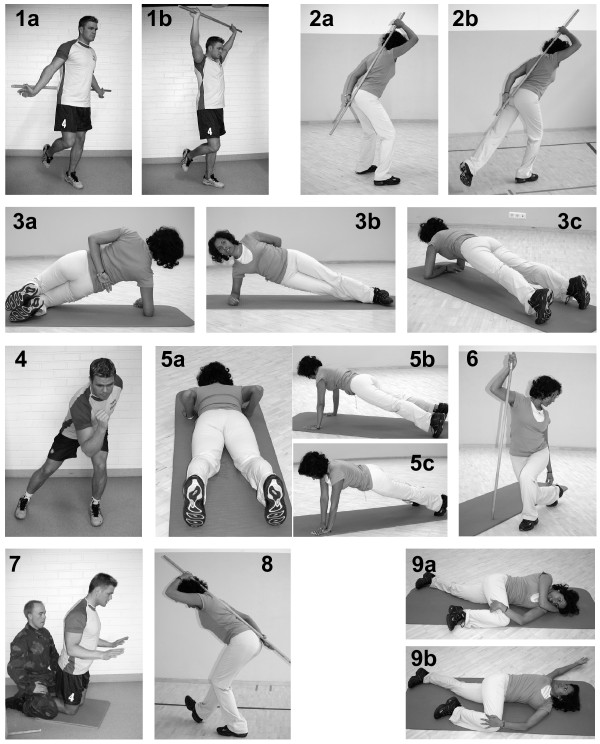
**Neuromuscular training exercises performed by the intervention group**. Exercises 1 through 9 and their specific aims are described in Table 2. The images were obtained in the Pori Brigade for the purposes of this study, and the individuals shown gave their consent to publish them.

The neuromuscular training programme was designed to enhance conscripts' movement control and agility, as well as to increase the stability of the trunk, knee and ankle. The focus of each of the nine exercises (see Figure 2) was on the use of proper technique, such as good posture, maintenance of core stability or positioning of the hips, knees and ankles, especially "knee over toe" position. Conscripts worked in pairs and were instructed to evaluate each other's technique and to provide feedback during training. The exercises and their repetitions are listed in Table 2 in the order of the exercises from one to nine. Two exercises (exercises 1 and 2) improved balance and posture, one exercise (exercise 4) improved coordination and agility, three exercises (exercises 2, 4 and 8) improved control of the lumbar neutral zone, two exercises (exercises 3 and 5) improved core (trunk) stability and endurance of the trunk muscles, one exercise (exercise 7) improved eccentric muscular work of the hamstring muscles, two exercises (exercises 6 and 8) improved the extensibility of the lower-extremity muscles and one exercise (exercise 9) improved the mobility of the thoracic spine. Exercises performed in upright positions (exercises 1, 2, 4, 6 and 8) followed the exercise principle of a closed kinetic chain [28].

**Table 2:** Neuromuscular training programme^a^

Exercises and repetitions	Aim
Exercise 1 One-leg standing with a stick 20 repetitions, 10 with each leg	Improvement in shoulder and neck posture and mobility Enhancement of balance and coordination
Exercise 2 Squat exercises with a stick using, respectively, two legs or one leg 16 repetitions on two legs 16 repetitions, eight each with one leg	Enhancement of control of lumbar NZ Increase in lower-extremity muscular strength Enhancement of balance
Exercise 3 Horizontal side support Stage 1 with flexed knees: five repetitions with 5 seconds of static holding on alternating sides (5 + 5) Stage 2 with straight knees: five circles from side to side with 5-second hold for each position (side, belly and side)	Enhancement of co-contraction of trunk muscles Improvement in lower-back and trunk stability Increase in trunk muscular endurance
Exercise 4 Jumping from side to side Rhythm: four slow jumps + eight fast jumps Exercise time: 60 seconds	Enhancement of coordination and agility Enhancement of control of lumbar NZ Increase in lower-extremity muscular endurance
Exercise 5 Modified pushups As many repetitions as possible Exercise time: 60 seconds	Improvement of upper-extremity extensor strength Enhancement of co-contraction of trunk muscles Improvement in lower-back and trunk stability
Exercise 6 Stretching exercise for hip flexor muscles 10-second stretch done five times on alternating sides	Increase in extensibility of hip flexor and side muscles Increase in lower-extremity muscular strength
Exercise 7 Hamstring exercise on the knees Eight to 12 repetitions	Increase in eccentric capacity of hamstring muscles Enhancement of trunk motor control
Exercise 8 Stretching exercise with a stick for hamstring muscles Three repetitions of 20-second stretches each with alternating legs	Increase in extensibility of hamstring and calf muscles Enhancement of control of lumbar NZ
Exercise 9 Upper-body rotation while lying on one's side; a "yoga stretch" Duration of 60 seconds for each side	Improvement in rotational mobility of thoracic spine Increase in extensibility of pectoral muscles

^a^NZ, neutral zone.

During the first 8 weeks of basic training, neuromuscular training was conducted three times weekly as part of normal compulsory service in the intervention companies. The conscripts trained inside in small groups (approximately 40 men per group) led by the two instructors mentioned above. One exercise session lasted from 30 to 45 minutes and included the above-described nine exercises at moderate intensity. At the beginning of training, the emphasis was on correct performance of the technique, and later the challenge level for balance and coordination, the number of repetitions and the exercise load were increased. Each conscript was provided with a training book named "FIRE", which included the rationale for each exercise and contained illustrations showing how to use the correct technique. A training log was attached to the book.

During the specialised military training period (weeks 9 to 17) and the team training period (weeks 18 to 26), conscripts in the intervention companies were instructed to continue to exercise on their own at least once weekly. To support this command, instructed training sessions were provided in the evenings during the conscripts' leisure time. The conscripts were commanded to meet the exercise instructors once weekly to have their exercise logs checked and to receive individual guidance on how to correctly perform the exercises as needed. Conscripts in leading positions guided neuromuscular exercises as part of compulsory physical training two to four times per month during this training period. Selected exercises were also performed outdoors during field service.

#### Injury prevention counselling

Educational counselling was used to increase conscripts' knowledge and awareness of musculoskeletal injuries during various training situations. Each conscript received a guidance booklet with information on situations and duties that were supposed to pose a high risk for injury. These included the training on uneven surfaces, landing from vehicles and lifting heavy materials. Furthermore, information on how to manage acute injuries was provided. A 1-hour lecture on these potentially hazardous training and combat actions was provided by one of the instructors in the middle of the basic training period. The counselling lecture was repeated once during the special military training period. Furthermore, the leaders of the companies and the exercise instructors addressed the potential hazards of field service when appropriate.

Conscripts in the control companies conducted their service as usual, except for their awareness of their role as a control group in the study. In addition, they filled in all the study questionnaires and participated in the baseline fitness test battery.

### Outcome measures

The primary outcome measure was an acute lower- or upper-limb injury that occurred during the 6-month military service. The severity of injuries was a secondary outcome measure of the study. In addition to injuries sustained during active service hours, injuries and disorders that occurred during conscripts' leisure time or on the way to or from the garrison for leave were also included in the study.

### Injury definition and registration

The data for the first cohort to arrive were collected from 10 July 2006 to 5 January 2007; for the second cohort that arrived, they were collected from 8 January 2007 to 6 July 2007; for the third cohort arrival, they were collected from 9 July 2007 to 4 January 2008; and for the fourth cohort to arrive, they were collected from 7 January 2008 to 4 July 2008. Injury was defined as an acute event that resulted in physical damage to the body for which the conscript sought medical care from the garrison clinic. Overuse, heat or cold injuries were not included in the analysis. During military service, all conscripts had to use the services of military healthcare units. The date, anatomical location, type, aetiological circumstances, severity and diagnosis of each injury were registered in computerised patient records. Because conscripts may have sought medical care several times for the same injury, the total number of health clinic visits exceeded the number of injuries. The health clinic visits were considered to be for the same injury when the conscript had sustained an injury of the same type and location during the preceding 2 weeks or if a physician had marked in the conscript's files that the reason for the visit was related to the previous injury.

The type of injury was categorised as acute if it had a sudden onset involving known trauma [19,20,29]. For example, sprains, strains, ligament ruptures and joint dislocations were categorised as acute injuries.

After careful clinical examination, necessary diagnostic tests and radiographs, the most accurate diagnosis was selected by a physician according to the *International Statistical Classification of Diseases and Related Health Problems, 10th Revision *[30]. The anatomical location of the injury was reported according to the diagnosis. The severity of the injury was categorised according to the number of days of limited duty, with 1 to 3 days being minimal, 4 to 7 days being mild, 8 to 28 days being moderate and more than 28 days being severe [19,20,31]. Limited duty involved a physical restriction that prevented the conscript from fully participating in military training events. Release from military service was indicated when a physician determined that a conscript was unable to continue military training. Releases from military service due to musculoskeletal injury were registered as severe injuries.

### Statistical analysis

SPSS 17.0 for Windows software (SPSS Inc., Chicago, IL, USA) was used for statistical analysis. All analyses were performed according to the intention-to-treat principle. The primary analysis was intervention group vs. control group for assessment of the difference of change in injury incidence between the prestudy period and the study period. Secondary analysis was performed to assess differences between participants at two fitness levels (low vs. moderate to high).

Injury incidence was calculated by dividing the number of new injuries by the exposure time. The incidences with 95% confidence intervals (95% CIs) were expressed per 1,000 person-days. To examine differences in injury rates between the intervention and control groups, the unadjusted and adjusted hazard ratios (HRs) between groups were obtained by using the Cox proportional hazard model for categorical outcomes and the negative binomial model for count data (number of off-duty days). The negative binomial model was chosen instead of the Poisson regression model because of the distribution of the count data. The overdispersion parameter was taken into account by estimating the value in the negative binomial model. *P *< 0.05 was considered statistically significant.

Results were expressed as HRs and calculated with 95% CIs with age at baseline forced into the model. The interaction term of company (intervention vs. control) and study period (prestudy period vs. study period) was entered into the model to analyse the differences in changes in incidence of injuries between intervention and control companies. In the data analysis, based on the published literature, conceptually compatible and logical risk factors were chosen for multivariate models. Only possibly significant explanatory variables (*P *< 0.20) in the initial univariate models were included for the multivariate conceptual models. Urbanisation level of the conscript's home residence was included in the multivariate model as a possible confounder. Higher age, smoking status (previous or current regular smoker), high alcohol intake, poor baseline medical condition (chronic impairment or disability due to prior musculoskeletal injury, as well as earlier musculoskeletal symptoms or orthopaedic surgery), poor school performance (educational level and grades combined) and high waist circumference were entered into the model as known or possible risk factors. Physical activity level during the 3 months before entering the military and the CPFI were considered effect modifiers and were entered into the multivariate model.

## Results

The details of the flow of participants through the study are shown in Figure 1. The rate of consent to participate was 98%. Most dropouts were due to a change of company after the 8-week basic military training period. Twenty dropouts in the intervention group and twenty-nine in the control group were due to musculoskeletal injuries. Data for these men who dropped out were included in the analyses for the time during which they participated. The intervention group's compliance was good. The intervention group followed the training programme according to the plan three times weekly as part of compulsory service during the first 8-week period. After this point, an average of 83% of the conscripts attended the training sessions and reached the preset minimum number of exercise sessions.

The number and incidence of acute injuries and corresponding HRs for men in the intervention and control companies during the prestudy and study periods are shown in Table 3. The intervention companies had a somewhat higher risk of injury before the intervention. In the intervention companies, the risk for acute ankle injuries decreased significantly compared to that of the control companies during the study period (adjusted HR = 0.34, 95% CI = 0.15 to 0.78, *P *= 0.011). The risk decline was observed in conscripts with a low baseline fitness level, as well as in those with a moderate to high baseline fitness level (Tables 4 and 5). In addition, among men with moderate to high baseline fitness, the risk for acute upper-extremity injury decreased significantly in the intervention companies compared to the control companies (adjusted HR = 0.37, 95% CI = 0.14 to 0.99, *P *= 0.047) (Table 4). Furthermore, the intervention companies tended to have less training time loss due to injuries (adjusted HR = 0.55, 95% CI = 0.29 to 1.04).

**Table 3:** Incidence per 1,000 person-days of different types of musculoskeletal injuries and hazard ratios for changes in incidence between the intervention and control companies during prestudy and study periods^a^

Variable	Company	Prestudy period (*n *= 508/436)^b^		Study period (*n *= 501/467)^b^		Age-adjusted HR (95% CI)	HR adjusted model^c^ (95% CI)
		Number	Incidence	Number	Incidence		
Acute injuries, all	Int	246	3.16	150	2.14	0.74 (0.52 to 1.06)	0.75 (0.51 to 1.09)
	Ctrl	149	2.73	155	2.44		
Lower extremity	Int	136	1.75	90	1.28	0.84 (0.55 to 1.30)	0.82 (0.52 to 1.31)
	Ctrl	91	1.67	96	1.51		
Knee	Int	50	0.64	48	0.68	1.05 (0.55 to 2.00)	1.32 (0.65 to 2.67)
	Ctrl	35	0.64	38	0.60		
Ankle	Int	37	0.48	17	0.24	0.38 (0.17 to 0.86)	0.34 (0.15 to 0.78)
	Ctrl	21	0.38	37	0.58		
Upper extremity	Int	53	0.68	31	0.44	0.57 (0.28 to 1.16)	0.52 (0.24 to 1.12)
	Ctrl	26	0.48	31	0.49		
Total number of off-duty days^d^	Int	917	11.8	546	7.8	0.46 (0.26 to 0.83)	0.55 (0.29 to 1.04)
	Ctrl	419	7.7	677	10.7		
Discharged from military service^e^	Int	34	0.44	42	0.60	0.78 (0.41 to 1.51)	0.81 (0.42 to 1.57)^f^
	Ctrl	26	0.48	52	0.82		
Follow-up days							
	Int	77,871		70,222			
	Ctrl	54,620		63,494			

^a^HR, hazard ratio; 95% CI, 95% confidence interval; Int, intervention company; Ctrl, control company. HRs were calculated by using the Cox proportional hazard model if not otherwise mentioned. Statistical significance level was set at *P *< 0.05. HRs are based on the interaction term of each study group (intervention or control), and study period was entered into the model to analyse the difference in the change in incidence between the groups. ^b^Number of conscripts in the intervention and control companies per study period; ^c^adjusted for age, urbanisation level of the home residence, smoking, alcohol intake, earlier musculoskeletal symptoms, orthopaedic surgeries, chronic disabilities due to earlier musculoskeletal injuries, school success, previous physical activity, waist circumference and conscript's physical fitness index (*n *= 11 adjusting variables); ^d^because of acute injuries, rate ratio was obtained using a negative binomial model; ^e^after the 2-week run-in period; ^f^not adjusted for waist circumference or physical fitness level, since 36 discharged individuals had missing information.

**Table 4:** Incidence per 1,000 person-days of different types of musculoskeletal injuries and hazard ratios for changes in incidence between the intervention and control companies during prestudy and study periods in moderately to highly fit conscripts^a,b^

Variable	Company	Prestudy period (*n *= 333/291)^c^		Study period (*n *= 315/298)^c^		Age-adjusted HR (95% CI)	HR adjusted model^d^ (95% CI)
		Number	Incidence	Number	Incidence		
Acute injuries, all	Int	160	3.05	85	1.88	0.77 (0.49 to 1.22)	0.74 (0.46 to 1.18)
	Ctrl	88	2.31	86	2.00		
Lower extremity	Int	82	1.56	56	1.24	0.88 (0.51 to 1.51)	0.82 (0.46 to 1.45)
	Ctrl	52	1.37	55	1.28		
Knee	Int	27	0.51	26	0.57	1.18 (0.51 to 2.75)	1.22 (0.49 to 3.01)
	Ctrl	22	0.58	21	0.49		
Ankle	Int	17	0.32	12	0.26	0.53 (0.18 to 1.51)	0.50 (0.17 to 1.46)
	Ctrl	12	0.32	20	0.46		
Upper extremity	Int	37	0.70	16	0.35	0.43 (0.17 to 1.09)	0.37 (0.14 to 0.99)
	Ctrl	15	0.39	20	0.46		
Total number of off-duty days^e^	Int	600	11.4	339	7.5	0.46 (0.22 to 0.97)	0.43 (0.19 to 0.97)^f^
	Ctrl	218	5.7	424	9.8		
Discharged from military service^g^	Int	10	0.19	19	0.42	1.06 (0.34 to 3.27)	1.13 (0.36 to 3.58)^f^
	Ctrl	8	0.21	20	0.46		
		Follow-up days					
	Int	52,542		45,316			
	Ctrl	38,052		43,054			

^a^HR, hazard ratio; 95% CI, 95% confidence interval; Int, intervention company; Ctrl, control company. HRs were calculated by using the Cox proportional hazard model if not otherwise mentioned. Statistical significance level was set at *P *< 0.05. HRs are based on the interaction term of each study group (intervention or control), and study period was entered into the model to analyse the difference in the change in incidence between the groups. ^b^Two highest tertiles of conscripts according to physical fitness (Conscript's physical fitness index > 14.04 points); ^c^number of conscripts in the intervention and control companies per study period; ^d^adjusted for age, urbanisation level of the home residence, smoking, alcohol intake, earlier musculoskeletal symptoms, orthopaedic surgeries, chronic disabilities due to earlier musculoskeletal injuries, school success, previous physical activity and waist circumference (*n *= 10 adjusting variables); ^e^because of acute injuries, rate ratio was obtained from negative binomial model; ^f^not adjusted for waist circumference, since 15 discharged individuals had missing information; ^g^after the 2-week run-in period.

**Table 5:** Incidence per 1,000 person-days of different types of musculoskeletal injuries and hazard ratios for change in incidence between the intervention and control companies during prestudy and study periods in low fitness conscripts^a,b^

Variable	Company	Prestudy period (*n *= 166/133)^c^		Study period (*n *= 174/144)^c^		Age-adjusted HR (95% CI)	HR adjusted model^d^ (95% CI)
		Number	Incidence	Number	Incidence		
Acute injuries, all	Int	83	3.37	60	2.47	0.77 (0.42 to 1.39)	0.79 (0.41 to 1.51)
	Ctrl	58	3.63	63	3.21		
Lower extremity	Int	53	2.15	32	1.32	0.84 (0.40 to 1.78)	0.86 (0.38 to 1.92)
	Ctrl	39	2.44	38	1.94		
Knee	Int	22	0.89	20	0.82	1.05 (0.37 to 2.99)	1.48 (0.46 to 4.81)
	Ctrl	13	0.81	14	0.71		
Ankle	Int	20	0.81	5	0.21	0.23 (0.06 to 0.85)	0.17 (0.04 to 0.68)
	Ctrl	9	0.56	17	0.87		
Upper extremity	Int	14	0.57	14	0.58	1.04 (0.30 to 3.62)	0.93 (0.24 to 3.56)
	Ctrl	10	0.63	8	0.41		
Total number of off-duty days^e^	Int	303	12.3	203	8.4	0.69 (0.26 to 1.82)	0.64 (0.23 to 1.79)^f^
	Ctrl	198	12.4	217	11.1		
Discharged from military service^g^	Int	17	0.69	13	0.54	0.68 (0.24 to 1.97)	0.72 (0.24 to 2.12)^f^
	Ctrl	11	0.69	15	0.76		
		Follow-up days					
	Int	24,599		24,292			
	Ctrl	15,963		19,628			

^a^HR, hazard ratio; 95% CI, 95% confidence interval; Int, intervention company; Ctrl, control company. HRs were calculated by using the Cox proportional hazard model if not otherwise mentioned. Statistical significance level was set at *P *< 0.05. HRs are based on the interaction term of each study group (intervention or control), and study period was entered into the model to analyse the difference in the change in incidence between the groups. ^b^The lowest tertile of conscripts according to physical fitness (conscript's physical fitness index ≤14.04 points); ^c^number of conscripts in the intervention and control companies per study period; ^d^adjusted for age, urbanisation level of the home residence, smoking, alcohol intake, earlier musculoskeletal symptoms, orthopaedic surgeries, chronic disabilities due to earlier musculoskeletal injuries, school success, previous physical activity and waist circumference (*n *= 10 adjusting variables); ^e^because of acute injuries, rate ratio obtained from negative binomial model; ^f^not adjusted by waist circumference, since 16 discharged individuals had missing information; ^g^after the 2-week run-in period.

## Discussion

The present study was a randomised, controlled trial designed to evaluate the effects of a neuromuscular training and injury prevention counselling programme on injury risk in a representative sample of young Finnish men. The training programme focused on improving the men's motor skills and body control. Compared to the control group, the intervention group had significantly fewer ankle injuries and a trend toward a decreased risk of upper-extremity injuries.

The present study has several strengths. First, the definition of injury was clear and predetermined. In addition, the data set of injuries was collected using computerised patient files. This guaranteed a high coverage of injuries because all patients who entered the garrison clinic were recorded in the computerised system. Second, the study design with unit randomisation included preplanned injury prevention counselling in the intervention group (attention effect) and resulted in minimal intervention influence on the control group (avoidance of contamination bias). Third, the participation rate was high (98%), and compliance with training was very good because of the army training setting. Fourth, the military environment provided highly standardised conditions for investigating the effect of the intervention: Conscripts in all cohorts in the trial trained in the same area, ate the same food and lived in the same barracks, and, moreover, the daily military programmes were nearly equal, providing equal opportunity for rest and sleep [32,33].

The study also has limitations. First, the lack of individual randomisation and the impossibility of full double blinding in this type of study limit the strength of the conclusions. The randomisation phase, data collection and data analysis were fully blinded, but for obvious reasons the young conscripts and exercise instructors could not be masked. Second, the group or cluster size was large because of the military setting, thus leading to a low number of allocated groups. Although this factor was taken into account in the study design and we were able to assess the baseline risk of injury in the companies during the prestudy period, the findings can be generalised only to similar settings in which young individuals are trained and counselled in groups or teams. Third, the findings can be generalised to young men only because no more than 3% of the conscripts were females, and they were excluded from the study. A fourth limitation is the fact that after the initial 8 weeks of basic training, the training programmes became more divergent as a result of the more specialised military service in each company. This also caused some participants to drop out because of a company change. On the other hand, all conscripts were followed up for the first 8 weeks of service. Finally, some conscripts might have been more inclined to seek professional medical care than others. This factor should have affected all of the companies similarly, however.

In the present study, a strong emphasis was placed on proper technical performance of every single exercise manoeuvre. Before the intervention the instructors were educated with regard to the correct training technique and how to best instruct each exercise and observe typical mistakes in each exercise manoeuvre, as well as how to appropriately correct mistakes. Some previous studies have indicated that neuromuscular training can play a crucial role in preventing acute lower-extremity injuries [12-17,19,20], and the present intervention study supports those findings. In the study of Hewett and co-workers [12], multiple 6-week training programmes for high school sports teams decreased the rate of serious knee ligament injuries as well as the rate of noncontact knee ligament injuries. The study of Olsen and colleagues [16] showed that a structured warmup programme among young handball players reduced the risk of traumatic knee and ankle injuries, as well as the overall risk for severe and noncontact injuries. In a recent randomised study of top-level pivoting sport athletes [19], we found significant reductions in the risk of ankle injuries. Soligard and colleagues [20] found that a comprehensive neuromuscular training programme was effective in decreasing overuse injuries among young soccer players.

One of the reasons for the current study was that at the turn of the millennium, there was a substantial (62%) rise in the number of premature discharges in the Finnish army due to musculoskeletal injuries [34]. This was most likely due to the 100% increase in physical exercise in the Finnish military service programme in July 1998. At that time, 8% to 10% of the conscripts were prematurely discharged from the Finnish Defence Forces. In a very recent study, we found that co-impairments in cardiorespiratory and muscular fitness (that is, poor results in Cooper's test combined with a poor result in the standing long jump, pushup or back-lift test) were highly associated with musculoskeletal injuries and disorders, showing a dose-response relationship. Similarly, abdominal obesity and high BMI were clearly associated with poor outcomes [35].

The present study underlines the importance of musculoskeletal injuries as a cause of morbidity and premature discharge from military service in the Finnish Defence Forces. Given that 90% of young men in Finland enter military service, the high occurrence of injuries in this population has a direct impact on public health. The current findings provide a challenge to researchers and military personnel to better recognise and identify the risk factors and mechanisms of injury to initiate preventive actions among conscripts.

## Conclusions

A neuromuscular training and injury prevention counselling programme was effective in preventing acute ankle and upper-extremity injuries in young male army conscripts. A similar programme could be useful for all young individuals who are initiating regular exercise.

## Competing interests

We declare that all authors had (1) no financial support for the submitted work from anyone other than their employer; (2) no financial relationships with commercial entities that might have an interest in the submitted work; (3) no spouses, partners or children with relationships with commercial entities that might have an interest in the submitted work; and (4) no nonfinancial interests that may be relevant to the submitted work.

## Authors' contributions

JP, HT, JS, VM, OO, PV, PK and HP contributed to study conception and design. JP and JS carried out the literature search and coordinated and managed all parts of the study, including testing and refining the intervention and data collection. OO and PV contributed to the testing of the intervention programme and the education of instructors, which were planned with JP and JS. HT conducted data collection and performed preliminary data preparation. HT conducted data analyses, and all of the authors contributed to the interpretation of data. JP wrote the first draft of the paper, and all authors provided substantive feedback on the paper and contributed to the final manuscript. All authors have approved the submitted version of the manuscript. HP is the guarantor.

## Pre-publication history

The pre-publication history for this paper can be accessed here:

http://www.biomedcentral.com/1741-7015/9/35/prepub
